# Antimicrobial Resistance Profile of* E. coli* Isolated from Raw Cow Milk and Fresh Fruit Juice in Mekelle, Tigray, Ethiopia

**DOI:** 10.1155/2018/8903142

**Published:** 2018-03-19

**Authors:** Haftay Abraha Tadesse, Netsenet Berhe Gidey, Kidane Workelule, Hagos Hailu, Seyfe Gidey, Abrha Bsrat, Habtamu Taddele

**Affiliations:** College of Veterinary Medicine, Mekelle University, P.O. Box 2084, Mekelle, Ethiopia

## Abstract

**Aim:**

Foodborne illnesses represent a public health problem in developed and developing countries. They cause great suffering and are transmitted directly or indirectly between animals and humans and circulate in the global environment*. E. coli* are among them, causing a major public health problem. The aim of this study was therefore to study the antimicrobial resistance profile of* E. coli* from raw cow milk and fruit juice.

**Materials and Methods:**

A cross-sectional study was conducted from October 2016 to June 2017 on 258 samples collected from milk shops (*n* = 86), dairy farms (*n* = 86), and fruit juice (*n* = 86) in different subcities of Mekelle. Bacteriological procedures were used for isolation of* E. coli *in the collected samples and for identification of the antimicrobial resistance profile.

**Result:**

The overall mean viable bacterial count and standard deviation of samples from milk shop, fruit juice, and dairy milk were found to be 8.86 ± 10^7^, 7.2 ± 10^7^, and 8.65 ± 10^7^ CFU/ml and 33.87 ± 10^6^, 6.68 ± 10^6^, and 22.0 ± 10^6^, respectively. Of the samples tested, 39 from milk shops (45.35%), 20 from fruit juice (23.26%), and 24 from dairy farms (27.91%) were found to be positive for* E. coli. *The isolated* E. coli* were highly resistant to ampicillin (70%), sulfamethoxazole-trimethoprim (60%), clindamycin (80%), erythromycin (60%), chloramphenicol (50%), and kanamycin (50%) and were found to be susceptible to some antibiotics like gentamicin (100%), norfloxacin (100%), tetracycline (60%), polymyxin B (90%), and ciprofloxacin (90%).

**Conclusion:**

The current study supports the finding that raw milk and fruit juice can be regarded as critical source of pathogenic* E. coli. *This supports the need for strict monitoring and the implementation of effective hygienic and biosecurity measures in the whole food chain of these products as well as a prudent use of antimicrobials.

## 1. Background

Foodborne illnesses are an important challenge to public health and cause significant economic problem in many countries [1]. The crucial goal of all food safety programs is to prevent food products contaminated by potential pathogens from reaching the consumer. Milk is an excellent medium for bacterial growth, which not only spoils the milk and associated products but also can cause infections in consumers [2]. Because of the specific production, it is not possible to fully avoid contamination of milk with microorganisms; therefore the microbial contamination of milk is an important tool in determining its quality [3, 4]. Huge numbers of microbes can get access to milk and various milk products including* E. coli*, which is an indicator of milk and fruit juice contamination, constituting a public health hazard [5].* E. coli* infection is a disease that can be transmitted directly or indirectly between animals and humans [6].

It is common in developing countries such as Ethiopia because of the prevailing poor food handling and sanitation practices, inadequate food safety laws, weak regulatory systems, lack of financial resources to invest in safer equipment, and lack of education for food handlers [7]. In countries where foodborne illness were investigated and documented, the relative importance of pathogens like* S. aureus*,* Campylobacter, E. coli*, and* Salmonella *species was recorded as a major cause [1, 8]. These organisms were known to cause acute gastroenteritis and may cause a more serious septicemic disease, usually in the very young, the elderly, or immunocompromised subjects [9, 10].

The ability of these microorganisms to survive under adverse conditions and to grow in the presence of low levels of nutrients and at suboptimal temperatures and pH values presents a formidable challenge to the agricultural and food-processing industries. The continued prominence of raw meats, eggs, dairy products, vegetable sprouts, fresh fruits, and fruit juices as the principal vehicles of human foodborne diseases poses a major challenge to coordinate sectorial control efforts within each industry [11]. Such juices have been found to be potential sources of bacterial pathogens, notably* Escherichia coli*,* Salmonella *spp.,* Shigella*, and* Staphylococcus aureus *[12].

Currently, the other major concern to human health is the issue of antimicrobial resistance due to use of antibiotics in livestock production as well as human diseases conditions in developing countries. In Ethiopia, the major antibiotics used for treatment of animal and human diseases include penicillin, streptomycin, gentamycin, and oxytetracycline. Even though it needs a better understanding of antibiotics use in Ethiopia, this resistance variation might be due to indiscriminate use of antimicrobials in animal production without prescription in the animal and human health sector, which might favor selection pressure that increased the advantage of maintaining resistance genes in bacteria [13]. So far, there are no studies conducted on the burden and drug sensitivity profile of* E. coli* in Mekelle city, Northern Ethiopia. In this study, we isolated* E. coli* and determined the drug resistance profile.

## 2. Materials and Methods

### 2.1. Study Area

The study was conducted from October 2016 to June 2017 in Mekelle city. Mekelle is the capital city of Tigray Regional State located about 783 km north of Addis Ababa, the capital city of Ethiopia, at geographical coordination of 39°28′ east longitude and 13°32′ north latitude. The average altitude of the city is 2300 m.a.s.l. with a mean annual rainfall and average annual temperature of 629 mm and 22°C, respectively [15]. The population of the city is 406,338 (195,605 males and 210,733 females) [15]. The city has seven subcities and 33* Kebeles* where over 139 juice houses, 48 dairy farms, and 123 milk shops (street vendor or retailer shops) are inhabited. Besides, the cities possess an extensive public transport network and active urban-rural exchange of goods with about 30,000 micro and small enterprises.

### 2.2. Study Design

A cross-sectional survey was conducted from October 2016 to June 2017 on raw cow milk and fresh fruit juice samples collected from different sources of raw milk shops, dairy milk supply centers, and juice houses in Mekelle. Purposive sampling technique was employed.

### 2.3. Research Methodology

*Sampling Technique and Collection.* There were a total of 258 food samples among which 172 were milk samples (86 from milk shops and 86 from dairy farms) and the remaining 86 are fresh juice samples (from 86 juice houses) in Mekelle city. After aseptic collection, samples were labeled and packed with sterile bottles and transported with an ice box to Microbiology and Public Health Laboratories, College of Veterinary Medicine, Mekelle University, for bacterial isolation. Samples were processed immediately for bacterial identification to species level using culture media and then isolates were kept in refrigerator at 4°C until microbial characterization with regular subculturing [16].

*Enumeration of Total Viable Count. *1 ml and gram of raw milk and fruit juice samples, respectively, were homogenized into 9 ml of serial peptone water/NSS and 10 g/1 g of each food item was weighed out and homogenized into 90 ml/9 ml of sterile distilled deionized water. Then serial dilutions were prepared. From the 10-fold dilutions of the homogenates, 1 ml of 10^−6^, 10^−7^ and 10^−8^ dilutions was cultured in replicate on standard plate count agar (HiMedia, India), using the pour plate method. The plates were then incubated at 37°C for 24 to 48 hrs. At the end of the incubation period, colonies were counted using the illuminated colony counter. The counts for each plate were expressed as colony-forming unit of the suspension (CFU/g) [17].

*Isolation and Characterization of Organism.* 1 ml and gram of thoroughly mixed raw milk and fruit juice sample, respectively, were aseptically added to 9 ml of sterile nutrient broth and incubated overnight at 37°C for 24 hours. The mixture of nutrient broth and raw milk and fruit juice sample was subcultured on sterile nutrient agar plate under aseptic condition and incubated at 37°C for 18–24 hours. Gram staining methods and further biochemical tests, catalase, carbohydrate utilization, indole production, citrate utilization, and methyl red tests, were carried out to identify the organisms that were isolated from the samples according to standard procedure described by [17, 18].

*Antimicrobial Susceptibility Test.* Antimicrobial susceptibility test, through Kirby diffusion test, was performed for all* E. coli *isolates following the protocol in [19]. At least 4-5 well-isolated colonies of the same morphological type are selected from a nonselective agar plate (nutrient agar); just the top of the colonies is touched and the growth transferred to a tube containing 4-5 ml of NSS or an equivalent medium such as peptone water broth. The inoculated broth is incubated at 35–37°C until a slight visible turbidity appears, usually within 2–8 hrs. The turbidity of the preincubated broth and the suspension of bacteria are adjusted by comparison with 0.5 McFarland turbidity standards. The standard and the test suspension are placed in similar 4–6 ml thin glass tubes or vials. The turbidity of the test suspension is adjusted with broth or saline and compared with turbidity standard against a white background with contrasting black lines, until the turbidity of the test suspension equals the turbidity standard [19].

The bacterial suspension was inoculated on to Mueller-Hinton agar (Oxoid, UK) with the sterile swab to cover the whole surface of the agar. The inoculated plates were left at room temperature to dry. Before using the antimicrobial disks, they were kept at room temperature for one hour and then dispended on the surface of media. Following this, the plates were incubated aerobically at 37°C for 24 hrs. The diameters of the zone of inhibition around the disks were measured to the nearest millimeter using calibrated rulers, and the isolates were classified as susceptible, intermediate, and resistant according to the interpretative accordance with the guidelines [20] (Table 1).

### 2.4. Data Management and Analysis

All data were checked against the standards and methods used to perform the study. Data was entered in Microsoft Excel spreadsheet and analyzed using STATA version 12. Descriptive statistics such as means, percentage, and frequencies were computed to report desired outputs. The antimicrobial resistance test was analyzed using WHONET software version 5 statistical package (http://www.who.int/medicines/areas/rational_use/AMR_WHONET_SOFTWARE/en/). Analysis of variance (ANOVA) was used to test the significant difference at *P* < 0.05.

## 3. Results

### 3.1. Total Viable Bacterial Count

The overall mean viable bacterial count recorded was 8.24 ± 10^7^. The individual sample type mean viable count and standard deviation of milk shop, fruit juice, and dairy milk are found in Table 2.

### 3.2. Isolation and Identification of* E. coli*

**Figure 1:**
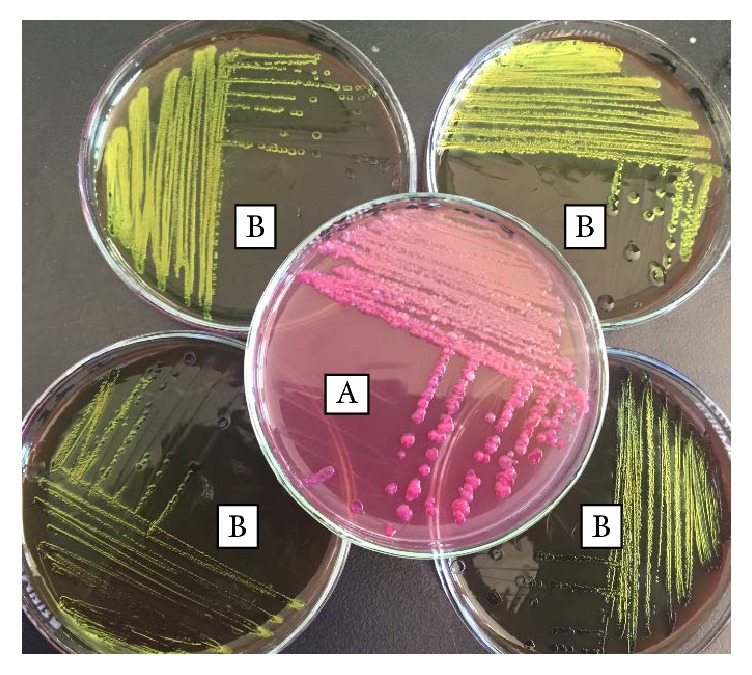
Colony observation on the two media. (A) MacConkey agar: smooth, circular pink colonies with spreading growth. (B) Eosin-methylene-blue agar: metallic sheen colony to growth.

Among the total 258 raw cow milk and fruit juice samples collected from different sources of Mekelle subcities, 115 (44.57%) samples were found to be positive for* E. coli*. Proportions of the isolation from milk shop, fruit juice, and dairy milk samples were indicated in Table 3. A statistically significant difference (*χ*^2^ = 20.4580; *P* value = 0.000) was recorded among samples from the three sites (Table 3 and Figure 1).

### 3.3. Antimicrobial Susceptibility Profile of* E. coli*

The antimicrobial resistance profiles of the bacterial isolates from raw cow milk and fruit juice samples were summarized in Table 4.* E. coli* showed resistance to antibiotics like ampicillin (70%), sulfamethoxazole-trimethoprim (60%), clindamycin (80%), erythromycin (60%), chloramphenicol (50%), and kanamycin (50%). The isolates were susceptible to some antibiotics like gentamicin (100%), norfloxacin (100%), tetracycline (60%), polymyxin B (90%), and ciprofloxacin (90%).

The multidrug resistance profile of the bacterial* E. coli* isolates is presented and the mean antibiotic sensitivity of* E. coli* species from raw milk shop, fruit juice, and dairy milk samples was found to be 16.16, 21.44, and 28.24, respectively (Table 5). In general, antimicrobial susceptibility test revealed that gentamicin, norfloxacin, polymyxin B, and ciprofloxacin were the antimicrobials indicated as active against* E. coli *isolated from this study.

A total of 13 multiple drug resistance patterns were observed. The highest MDR noted was AMP and STR (100%, 1/1). The maximum multiple drug resistance registered was resistance to one and three antibiotics with the combination AMP and STR, AMP STR ERY (Table 6).

## 4. Discussion

The current finding indicated that samples from milk shop, fruit juice, and dairy milk were found with a viable bacterial count load of 8.86 ± 10^7^, 7.2 ± 10^7^, and 8.65 ± 10^7^, CFU/ml, respectively, with an overall mean viable bacterial count of 8.24 ± 10^7^ CFU/ml. The highest mean value of microbial load (8.86 ± 10^7^ CFU/ml) was found from milk shop samples.

The current study showed a higher viable bacterial count than previous reports such as viable bacterial count from fresh fruit juice samples in Ethiopia [21] to raw milks for which a count was available, 96.8% ± 10^2^ CFU/ml, and raw milk cheeses for which a count was available, 98.6% ± 10^4^ CFU/g [22].

This variation could be due to hygiene difference, personal awareness, and proper handling of containers and the food itself. Furthermore, viable bacterial counts of 3.93 ± 0.01 CFU/ml [23] in milk samples from dairy farms in Khartoum State (Sudan) and 3.64 ± 0.776 CFU/ml 5 [24] from raw milk samples were reported in Ethiopia.

In the present study, 115 out of 258 (44.57%) samples were found to be positive for* E.coli*, of which 55 (63.95%) were from milk shop, 27 (31.40%) from fruit juice, and 33 (38.37%) from dairy farms. The result showed a high contamination rate, which might be attributed to poor hygienic sanitation. Statistically significant difference (*P* < 0.05) among the sample types in the prevalence of* E. coli* was recorded. A similar report was also made by previous researchers in Ethiopia. Other researchers reported higher* E. coli* isolates in raw milk value chain from farmers (89.74%) and shops (90.0%) in Arusha, Tanzania [25].

The isolation rate of* E. coli* in the present study was found to be lower (44.57%) compared to other reports such as those in Tanga, Tanzania, 100% [26], in Arusha, Tanzania, 90.67% [25], in Dar es Salaam, Tanzania, 83% [27], raw milk along chain, in Tando Jam, Pakistan, 51.66% [28] from milk vending shops, and 58% [29] from raw cow's milk in Ethiopia, whereas it was higher compared to other reports in Ethiopia, 26.6% [30], from milk sample from cafeteria.

The variation could be due to the reason that even when drawn under aseptic condition, milk always contains microorganisms that are derived from the milk ducts in the udder. In addition, contaminants coming from milking utensils, human handlers, unclean environmental conditions, and poor udder preparation might expose raw milk to bacterial contamination.

Antimicrobial resistance emerges from the use of antimicrobials in animals and human and the subsequent transfer of resistance genes and bacteria among animals, humans, animal products, and the environment. In Ethiopia, there have been reports on the drug resistance of* E. coli *isolates from animal-derived food products [31, 32]. The highest drug resistance recorded in the current study might be due to high antimicrobial use in dairy farms, fruit juices, and individual cows to treat various diseases affecting the dairy sector. Similarly, several studies have indicated that* E. coli* isolated showed high resistance to erythromycin (100%), streptomycin (50%), tetracycline (75%), and ampicillin (50%) and high sensitivity to penicillin (100%), gentamicin (75%), chloramphenicol (75%), and amoxicillin (50%) reported by [21] in Ethiopia.

Different researchers reported antimicrobial resistance of* E. coli* isolates of raw milk in their previous studies from Ethiopia. Reports from other researchers had also indicated* E. coli* isolates' resistance to ampicillin and cephalothin (84.6%), chloramphenicol (83.3%), tetracycline (88.9%), and gentamicin (65.9%) reported by [30] in Tigray, Ethiopia.

**Figure 2:**
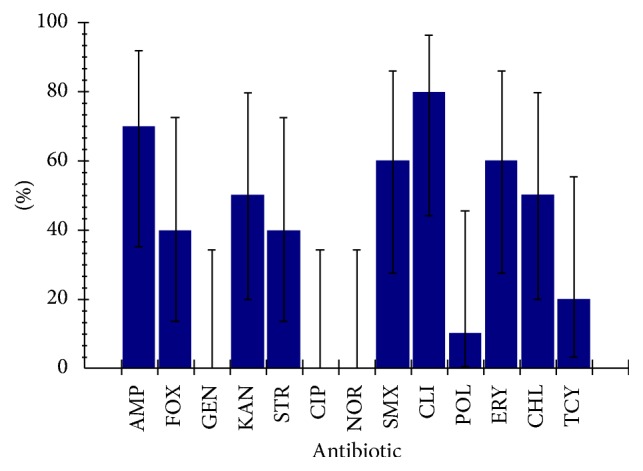
Antibiotic resistance profile of* E. coli* isolated from different samples.

Antibiotic resistance development among the bacteria poses a problem of concern. In all food samples in the present study,* E. coli *showed high resistance rates to ampicillin (70%), sulfamethoxazole-trimethoprim (60%), clindamycin (80%), erythromycin (60%), chloramphenicol (50%), and kanamycin (50%) and susceptibility to some antibiotics like gentamicin (100%), norfloxacin (100%), tetracycline (60%), polymyxin B (90%), and ciprofloxacin (90%) (Figure 2). The results of this study are in line with the findings of other studies conducted in different parts of the world [33, 34]. However, antimicrobial resistance rates obtained in this study were higher as compared to susceptibility patterns reported from previous studies [35–37].

**Figure 3:**
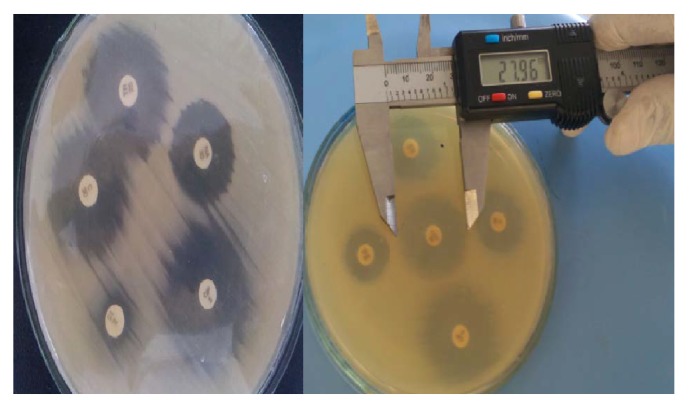
Antibiotic resistance profile and how to measure the inhibition zone of* E. coli* isolated from different samples.

*E. coli *isolates were sensitive to gentamicin, norfloxacin, tetracycline, polymyxin B, and ciprofloxacin (Figure 3). Similar studies conducted in Ethiopia by [38] and in Nigeria by [39] have reported comparable susceptibility rates. In this study, gentamicin, norfloxacin, tetracycline, polymyxin B, and ciprofloxacin were found to be the most effective antimicrobials against* E. coli *isolates. Furthermore, in this study, a high rate of multiple antimicrobial resistance (100%) was recorded, which is consistent with the reports of studies done elsewhere by other scholars [40, 41]. Increases in rate of resistance to different antimicrobials have been reported from previous studies conducted in different parts of the world [40, 41]. The remarkable degree of resistance to many drugs represents public health hazard due to the fact that foodborne outbreaks would be difficult to treat and this pool of MDR* E. coli* in food supply represents a reservoir for communicable resistant genes. Hence, due to the relatively limited access and high price to get the newly developed cephalosporin and quinolone drugs, the reports of prevalence of antimicrobial-resistant* E. coli* to relatively low-priced and regularly available antibiotics are alarming for a low-income society living in most developing countries, like Ethiopia.

## 5. Conclusion

The current study gives insights into the magnitude and incidence of* E. coli* from raw cow milk and fresh fruit juice samples. The study revealed that the development of antibiotic resistance against* E. coli* could pose serious threat for consumers in the study area. Hence, attention should be given to proper handling of the food items and using recent antibiotics in the treatment of diseases both in humans and in animals.

## Figures and Tables

**Table 1:** Drug sensitivity interpretive zone of inhibition diameters.

Antibiotics	Disc code	Potency	Zone of diameter		
			*S*	*M*	*R*
Erythromycin	ERY	15 *μ*g	≥23	14–22	≤13
Cephalothin	CF	30 *μ*g	≥18	15–17	≤14
Norfloxacin	f	50 *μ*g	≥17	13–16	≤12
Sulfamethoxazole-trimethoprim	SXT-TMP	300 *μ*g	≥16	11–15	≤10
Streptomycin	S	10 *μ*g	≥15	12–14	≤11
Kanamycin	KAN	30 *μ*g	≥18	14–17	≤13
Chloramphenicol	CHL	30 *μ*g	≥18		≤18
Tetracycline	TE	30 *μ*g	≥22	19–21	≤19
Gentamicin	GM	10 *μ*g	≥18	-	≤18
Ampicillin	AMP	10 *μ*g	≥15	12–14	≤11
Ciprofloxacin	CIP	5 *μ*g	≥20		≤20
Ceftriaxone	CRO	30 *μ*g	≥23	20–22	≤19
Clindamycin	CC	10 *μ*g	≥21	15–20	≤14

Source: [14]. R: resistant; I: intermediate; S: sensitive.

**Table 2:** Total mean viable bacterial count for different sample types.

Sample type	Mean bacterial count	±SD	Minimum bacterial count	Maximum bacterial count
Milk shop	8.86 ± 10^7^	33.87 ± 10^6^	1.5 ± 10^7^	1.25 ± 10^8^
Fruit juice	7.2 ± 10^7^	6.68 ± 10^6^	6.37 ± 10^7^	8.5 ± 10^7^
Dairy milk	8.65 ± 10^7^	22.0 ± 10^6^	6.4 ± 10^7^	1.23 ± 10^8^

Total	8.24 ± 10^7^	23.8 ± 10^6^	1.5 ± 10^7^	1.25 ± 10^8^

SD: standard deviation.

**Table 3:** *E. coli* from raw cow milk and fruit juice samples.

Sample type	Number of positive (%)	*χ*^2^	*P* value
Milk shop (*n* = 86)	55 (63.95)	20.4580	0.000
Fruit juice (*n* = 86)	27 (31.40)		
Dairy milk (*n* = 86)	33 (38.37)		

Overall (*n* = 258)	115 (44.57)		

**Table 4:** Antimicrobial resistance of *E. coli* isolated from raw milk and fruit juice sample.

Antibiotic	% resistant	% intermediate	% susceptibility	% resistance at 95% CI
Ampicillin	70	0	30	35.4–91.9
Cefoxitin	40	30	30	13.7–72.6
Gentamicin	0	0	100	0.0–34.5
Kanamycin	50	10	40	20.1–79.9
Streptomycin	40	30	30	13.7–72.6
Ciprofloxacin	0	10	90	0.0–34.5
Norfloxacin	0	0	100	0.0–34.5
Sulfamethoxazole	60	20	20	27.4–86.3
Clindamycin	80	10	10	44.2–96.5
Polymyxin B	10	0	90	0.5–45.9
Erythromycin	60	10	30	27.4–86.3
Chloramphenicol	50	10	40	20.1–79.9
Tetracycline	20	20	60	3.5–55.8

**Table 5:** Multidrug resistance of *E. coli* isolated from raw cow milk and fruit Juices sample.

Antimicrobial resistance	Antimicrobial	Isolates (%)
One	AMP (1)	(100%, 1/1)
	STR (1)	(100%, 1/1)
	AMP, STR, ERY (1)	(33%, 1/3)
	CHL, CIP, TCY, ERY (1)	(25%, 1/4)
Two	CHL, AMP, STR, ERY (2)	(25%, 2/8)

Five	CHL, AMP, STR, TCY, ERY (3)	(20%, 3/15)

AMP: ampicillin; ERY: erythromycin; CHL: chloramphenicol; CIP: ciprofloxacin; STR: streptomycin; TCY: tetracycline.

**Table 6:** In vitro antimicrobial sensitivity of *E. coli* isolated from different samples.

Sample type	Obs.	Mean	±SD	Min.	Max.
Milk shop	25	16.16	3.10	11	22
Fruit juice	25	21.44	2.81	13	26
Dairy milk	25	28.24	3.95	22	42

SD: standard deviation; Obs.: observation.

## Abbreviations

AMP: Ampicillin
ANOVA: Analysis of variance
CHL: Chloramphenicol
CIP: Ciprofloxacin
ERY: Erythromycin
I: Intermediate
MDR: Multiple drug resistance
NSS: Normal saline solution
R: Resistant
S: Sensitive
SD: Standard deviation
STR: Streptomycin
TCY: Tetracycline.
